# Evaluation of liver fibrosis with a monoexponential model of intravoxel incoherent motion magnetic resonance imaging

**DOI:** 10.18632/oncotarget.24758

**Published:** 2018-05-15

**Authors:** Cuiyun Chen, Fangfang Fu, Jing Zhang, Fangfang Guo, Meiyun Wang, Shaocheng Zhu, Dapeng Shi, Yuwei Tian

**Affiliations:** ^1^ Department of Radiology, Zhengzhou University People’s Hospital, Zhengzhou, Henan 450003, China; ^2^ Department of Hepatobiliary Surgery, Zhengzhou University People’s Hospital, Zhengzhou, Henan 450003, China; ^3^ Department of Pathology, Zhengzhou University People’s Hospital, Zhengzhou, Henan 450003, China

**Keywords:** liver fibrosis, IVIM-DWI, magnetic resonance imaging, monoexponential model

## Abstract

To evaluate hepatic fibrosis with a monoexponential model of intravoxel incoherent motion magnetic resonance imaging, and assess the potential application value of intravoxel incoherent motion (IVIM) in diffusion-weighted imaging (IVIM-DWI) in determining staging of liver fibrosis. 28 patients with hepatic fibrosis and 25 volunteers with healthy livers had IVIM examination and conventional MRI. All standard apparent diffusion coefficient (ADC) values of IVIM raw data were post-processed off-line after completion of data collection. All regions of interest (ROIs) were manually positioned by two experienced radiologists. All values of the different fibrosis stages in the study group were compared using independent sample *t* tests. Using ROC analysis, both AUC values of ADC_total_ and ADC_0-400-600-800_ from study and control group were found to be between 0.8 and 1 for staging fibrosis. The mean ADC_total_ and ADC_0-400-600-800_ values of the liver in the study group were significantly lower than the values in the control group (*P* < 0.05). Spearman rho correlation analysis was used to determine the relationship among fibrosis stages and the ADC_total_ and ADC_0-400-600-800_ in the study group. As the stage of the fibrosis increased, the values decreased. Significant differences between the two subgroups of liver fibrosis stages were found (*P* < 0.05). The monoexponential model of IVIM-DWI adopted multiple *b* values for quantitative analysis of the water molecules diffused in the tissue. It could be used as a noninvasive and valuable method for assessment of liver fibrosis.

## INTRODUCTION

Liver fibrosis is an important pathological feature of patients with chronic liver disease. It is also a serious health problem worldwide. Currently, there is no effective therapy available for liver fibrosis except the removal of underlying etiology or liver transplantation [1–3]. However, recent studies indicate that liver fibrosis is reversible, thus it is extremely important to accurately evaluate the degree of liver fibrosis and to make timely intervention measures, in order to prevent or reverse hepatic fibrosis process [4, 5]. At present, the gold standard for evaluating the degree of liver fibrosis is liver biopsy of a percutaneous puncture Liver biopsy is an invasive operation, patients are less widely accepted and the repeatability is poor [6].

With the development of echo-planar imaging (EPI) in magnetic resonance imaging plane echo technology, intravoxel incoherent motion in diffusion weighted imaging (IVIM-DWI) technology can be applied to the abdomen to evaluate metabolic and function changes before the morphology of liver fibrosis changes [7]. Recently, IVIM-DWI has been suggested as a new method for noninvasive diagnosis and staging of liver fibrosis [8, 9]. The purpose of this study is to evaluate hepatic fibrosis with a monoexponential model of intravoxel incoherent motion magnetic resonance imaging, and to assess the potential application value of IVIM-DWI in staging liver fibrosis. Our results showed that the monoexponential model of IVIM-DWI adopted multiple *b* values for quantitative analysis of the water molecules diffused in the tissue and that it could be used as a valuable noninvasive method for assessing liver fibrosis.

## RESULTS

### Patient information analysis

**Figure 1:**
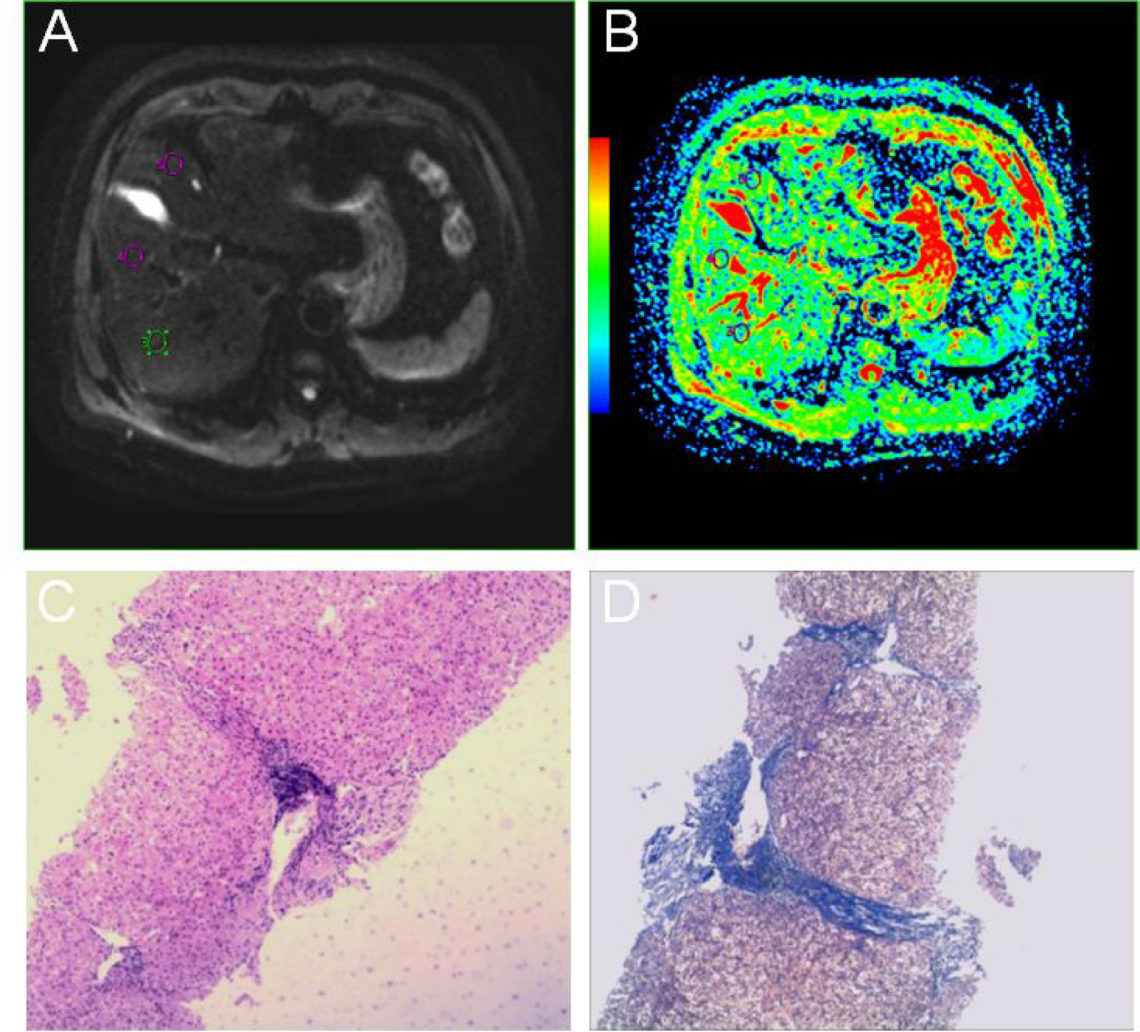
A 40-year-old male patient with a history of hepatitis (**A**, **B**) Multiple b DWIs, standard ADC. (**C**, **D**) HE staining with microscopic observation (×40) and Masson staining with microscopic observation (×40), respectively.

The fibrosis stages and histological activity grades of the 38 cases are presented in Table 1. Among the 38 patients, the fibrosis stage distributions are as follows: F0 group included 10 patients who were randomly selected from normal liver control group. F1 group included 4 patients who had mild histological activity. F2 group included 9 patients who had moderate histological activity. F3 group included 11 patients who had advanced histological activity. F4 group included 4 patients who had severe histological activity. All the standard ADC values of IVIM raw data were post-processed off-line at an ADW4.5 workstation (GE, USA) after completion of data collection (Figure 1A and 1B). Descriptive statistics, the LSD method of multiple comparison of variance for the study group and the control group were used. The mean ADC_total_, ADC_0-400-600-800_ values among the right posterior hepatic lobe, right anterior hepatic lobe and medial segment of the left lobe of the liver with fibrosis and the healthy livers had no significant differences (*P* > 0.05) (Table 2).

**Table 1:** Distribution of various stages of fibrosis with biochemical markers of liver function

Fibrosis Stage	ALT (7–40) U/L	AST (13–35) U/L	TP (65–85) g/L	ALB (40–55) g/L	TBIL (5–21) μM/L
F1(Mild fibrosis)	65	33	75.1	45.5	12.2
F1(Mild fibrosis)	38	25	70.4	44.7	9.2
F1(Mild fibrosis)	56	45	66.3	44.7	16.9
F1(Mild fibrosis)	23	27	66.3	42.9	10.3
F2(Moderate fibrosis)	27	28	83.2	52	9.3
F2(Moderate fibrosis)	32	16	73.1	45.1	7.8
F2(Moderate fibrosis)	60	40	86.4	46.8	21.6
F2(Moderate fibrosis)	86	81	75.5	44.9	8.7
F2(Moderate fibrosis)	42	38	69.3	38.6	10.1
F2(Moderate fibrosis)	33	26	72.2	41.2	11.2
F2(Moderate fibrosis)	49	36	66.8	48	6.9
F2(Moderate fibrosis)	33	31	74.2	41.3	18.9
F2(Moderate fibrosis)	38	28	78.2	38.7	9.2
F3(Advanced fibrosis)	26	27	80.6	45.9	16.7
F3(Advanced fibrosis)	54	40	81	45.7	10.3
F3(Advanced fibrosis)	47	37	60.2	34.9	17.1
F3(Advanced fibrosis)	57	29	68.5	40.3	15.9
F3(Advanced fibrosis)	69	58	59.6	32.7	18.7
F3(Advanced fibrosis)	73	69	69.3	42.1	15.4
F3(Advanced fibrosis)	63	49	70	49.3	13.1
F3(Advanced fibrosis)	72	63	57.2	33.6	18.7
F3(Advanced fibrosis)	69	45	66.4	39.2	12.6
F3(Advanced fibrosis)	60	51	68.2	40.5	12.9
F3(Advanced fibrosis)	49	32	60.3	39.3	10.6
F4(Cirrhosis)	53	43	70.9	37.8	13.4
F4(Cirrhosis)	57	49	69.5	39.2	15.3
F4(Cirrhosis)	60	55	70.3	38.4	16.1
F4(Cirrhosis)	58	50	69.6	39	14.7

**Table 2:** Multiple comparison analysis of variance among NL and LF segments

Parameters	RP	RA	LM	*P* values from multiple comparisons
	Mean ± SD (×10^–3^mm^2^/s)	Mean ± SD (×10^–3^mm^2^/s)	Mean ± SD (×10^–3^ mm^2^/s)	
NL-ADC_total_	1.36 ± 0.29	1.31 ± 0.21	1.40 ± 0.22	0.538^*^, 0.241^†^, 0.576^‡^
NL-ADC_0-400-600-800_	1.23 ± 0.04	1.22 ± 0.04	1.22 ± 0.03	0.179^*^, 1.000^†^, 0.179^‡^
LF-ADC_total_	1.10 ± 0.17	1.10 ± 0.27	1.10 ± 0.26	0.952^*^, 0.971^†^, 0.981^‡^
LF-ADC_0-400-600-800_	1.04 ± 0.15	1.02 ± 0.18	1.03 ± 0.17	0.582^*^, 0.859^†^, 0.709^‡^

RP = right posterior hepatic lobe; RA = right anterior hepatic lobe; LM = medial segment of the left lobe; NL = normal liver; LF = liver fibrosis; ^*^= RP vs RA; ^†^= RA vs LM; ^‡^= RP vs LM.

### ROC analysis

**Figure 2:**
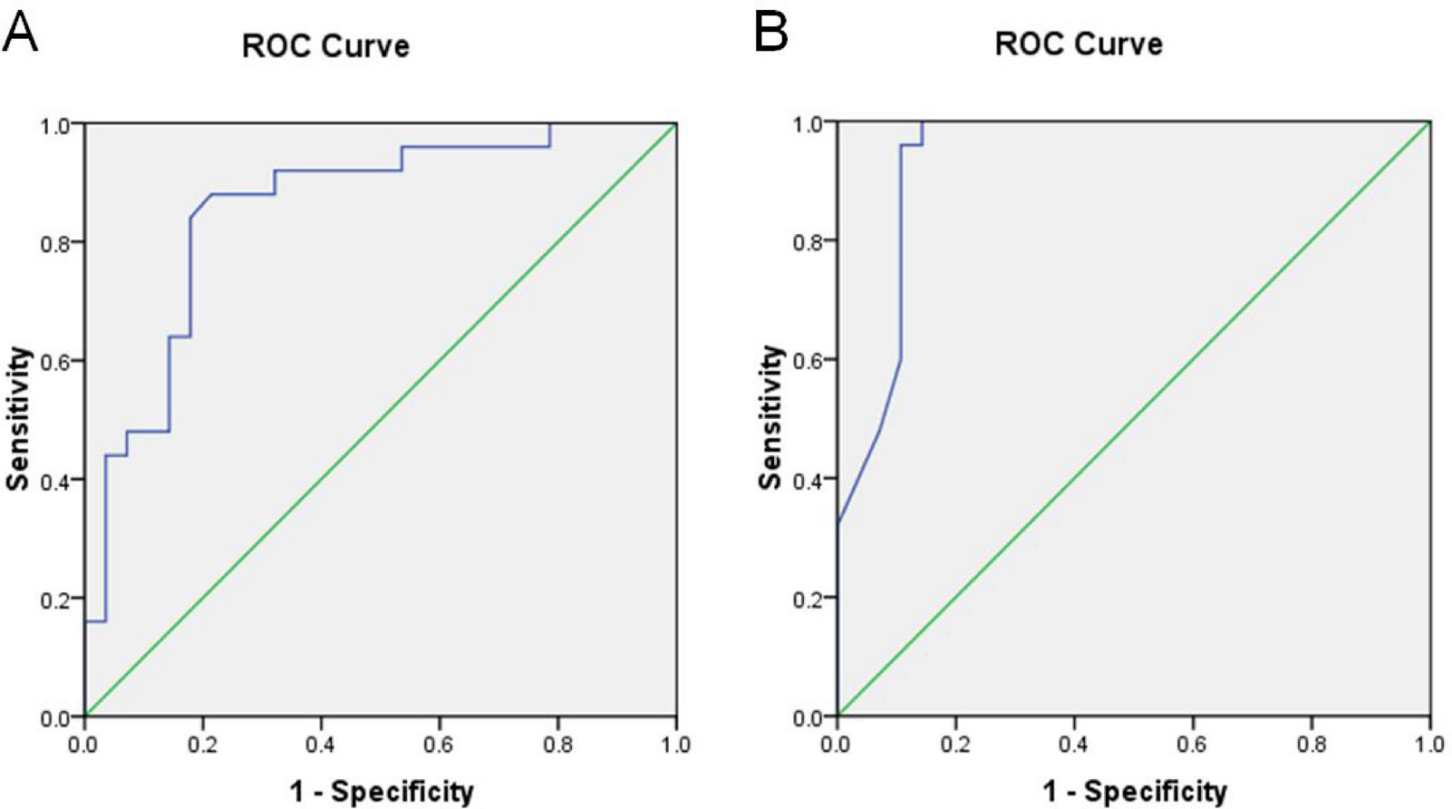
Results of receiver operator characteristic (ROC) analysis for study and control groups (**A**) ADC_total_. (**B**) ADC_0-400-600-800_.

Using ROC analysis, both AUC values of ADC_total_ and ADC_0-400-600-800_ from all data of the study and control groups were between 0.8 and 1 for staging fibrosis (Figure 2A and 2B). Results of the ROC analysis are also summarized in Table 3.

**Table 3:** ROC analysis of ADC_total_ and ADC_0-400-600-800_ for the diagnosis of fibrosis

Parameters	ADC_total_	ADC_0-400-600-800_
AUC	0.855	0.939
95% CI	0.750–0.960	0.871–1.007
Cutoff values	1.240	1.173
Sensitivity (%)	88	100
Specificity (%)	78.6	85.7
PPV (%)	80.4	87.5
NPV (%)	86.8	100

### IVIM lowers ADC_total_ and ADC_0-400-600-800_ values

Descriptive statistics, the independent samples *t* test for the study and control groups were used. The mean ADC_total_ and ADC_0-400-600-800_ values of the liver in the study group were significantly lower than those values in the control group (*P* < 0.05). The results are summarized in Table 4.

**Table 4:** Comparison of the mean ADC_total_ and ADC_0-400-600-800_ values

Parameters	Control Group (×10^–3^ mm^2^/s)	95% CI	Study Group (×10^–3^ mm^2^/s)		95% CI	*P* value by *t* test
ADC_total_ ADC_0-400-600-800_	1.36 ± 0.16 1.22 ± 0.02	1.29–1.42 1.21–1.23		1.10 ± 0.20 1.03 ± 0.16	1.02–1.17 0.96–1.09	0.000 0.000

### IVIM determines the relationship between ADC values and fibrosis stages

Spearman rho correlation analysis was used to determine the relationship among fibrosis stages and the ADC_total_ and ADC_0-400-600-800_ in the study group. As the stage of fibrosis progressed, the values decreased. The independent samples *t* test was used to compare the mean values (ADC_total_ and ADC_0-400-600-800_) of liver fibrosis stages between the subgroups F0-1 and F2-4, the subgroups F0-2 and F3-4, the subgroups F0-3 and F4. We found significant differences between the three subgroups of liver fibrosis stages (*P* < 0.05). The results are summarized in Table 5.

**Table 5:** Comparison of mean values between fibrosis stages (*t* test)

	F0-1 VS F2-4	values		F0-2 VS F3-4	values		F0-3 VS F4	values	
Parameters	Mean ± SD (×10^–3^mm^2^/s)	Mean ± SD (×10^–3^mm^2^/s)	*P* values	Mean ± SD (×10^–3^mm^2^/s)	Mean ± SD (×10^–3^mm^2^/s)	*P* values	Mean ± SD (×10^–3^mm^2^/s)	Mean ± SD (×10^–3^mm^2^/s)	*P* values
ADC_total_	1.34 ± 0.20	1.08 ± 0.19	0.001	1.27 ± 0.20	1.04 ± 0.20	0.002	1.22 ± 0.19	0.84 ± 0.21	0.001
ADC_0-400-600-800_	1.20 ± 0.08	1.01 ± 0.17	0.000	1.16 ± 0.09	0.95 ± 0.18	0.000	1.13 ± 0.09	0.68 ± 0.14	0.000

## DISCUSSION

Liver fibrosis is an early pathological indicator of chronic liver disease. It is an imbalance between fiber formation and degradation in the progression of all kinds of chronic liver diseases. Excessive quantities of collagen deposits in the liver can gradually change the stage of liver cirrhosis and even lead to hepatocellular carcinoma (HCC) [1–3, 19]. The progression of liver fibrosis can be prevented from further development by clinical treatment. If it is not timely treated, it may progress to the decompensation period of cirrhosis, even with all kinds of complications of end-stage liver disease.

Early treatment can obviously improve the prognosis. At present, early diagnosis of hepatic fibrosis and determination of its stage is crucial for treatment. Although percutaneous liver biopsy is considered the gold standard diagnostic approach, it has several significant drawbacks. It is an invasive procedure not well accepted by patients due to the high cost of inspection, sampling bias, poor repeatability and complications. Thus, liver biopsy is not used as a routine screening method for the dynamic detection of liver fibrosis progression and treatment. In addition, when using conventional MR imaging at the stage of liver fibrosis, it is hard to find slight abnormalities or the screenings are unable to display very slight abnormal signals. Clinically, patients with liver fibrosis often had asymptomatic or mild abdominal distension under conventional MR imaging, rendering conventional MR imaging not as useful for definitive classification in liver fibrosis diagnosis [20]. The purpose of this study is to investigate the application value of the monoexponential model of IVIM-DWI in evaluation of liver fibrosis and staging, given that this technology is a new and noninvasive method that uses no contrast agent, and has good maneuverability and repeatability of inspection.

Standard ADC (the unit is mm^2^/s) is the traditional ADC monoexponential model fitting b0 data and threshold value of DWI data, which can get b or higher dispersion coefficient. Its basic principle is the same as the traditional ADC monoexponential model, reflecting the movement of water molecules within the organ. ADC values are the most common quantitative indicators. The calculating formula is Sb/S0 = exp (bADC). S0 and Sb represent the low and high *b* signal value of DWI, respectively. The ADC values reflect the speed of the water molecules. The speed of the dispersion indicates the ADC values. The ADC value is influenced by water molecules’ diffuse and capillary perfusion, which is closely related to its *b* values. If the *b* value is >200 s/mm^2^, the result mainly reflects the dispersion of the water molecules; if the *b* value is <200 s/mm^2^, the result mainly reflects the blood perfusion of the situation. In this study, the standard ADC values (namely ADC_total_) by 7 *b* values and (namely ADC_0-400-600-800_) by 4 *b* values were according to the formula of Sb/S0=exp (bADC), respectively. Thus, it is easy to understand from the formula. While ADC values are not only reflected by distribution of the *b* value (> 200 s/mm^2^ or < 200 s/mm^2^), they can be simultaneously affected by water molecules’ diffuse and capillary perfusion. Since ADC values were related to the number of *b* values, the more the values of b that were chosen, the more objective and accurate were the measured results. Using ROC curve analysis, we concluded that the mean values of ADC_total_ and ADC_0-400-00-800_ for diagnosis of liver fibrosis by area under the ROC curve were 0.855 and 0.939, respectively. ADC_0-400-00-800_ showed better diagnosis ability than ADC_total_ in the diagnosis of liver fibrosis. When taking a certain critical value of diagnosis, the results showed sensitivity, specificity, false positives and false negatives of ADC_0-400-00-800_ to be higher than ADC_total_.

Liver fibrosis is the main collagen in the formation of the extracellular matrix (ECM) caused by excessive deposition, which results in significant proton decreases [21, 22]. Therefore, if it has fibrotic distortion within the hepatic lobule of organ’s structure, the collagen deposition of the extracellular matrix will limit the movement of water molecules. The result can lead to the lower ADC values of liver fibrosis and liver cirrhosis, rather than the higher values found in a normal liver [23–26]. According to reports in the literature [27], experimenters chose a single *b* value of >200 s/mm^2^ in traditional DWI–MRI which could mainly reflect water molecules diffused in the organ. The ability of DWI–MRI in diagnosing liver fibrosis was only reached at a medium level. However, in recent years, increasing number of reports in the literature adopted multiple *b* values, which were then compared to different *b* values in the diagnosis of liver fibrosis. Vaziri-Bozorg *et al.* [13] selected 11 cases with normal livers as the control group and 33 patients with hepatitis B or C as the study group, using the *b* values of 0–500, 0–700, 0–1000 s/mm^2^. The results demonstrated that using the *b* value of 500 s/mm^2^ in the diagnosis of liver fibrosis was significantly better than *b* values of 700 s/mm^2^ and 1000 s/mm^2^. Ozkurt H *et al.* [28] studied 24 patients with liver fibrosis and 22 cases with healthy subjects. The *b* values were set at 250 s/mm^2^, 500 s/mm^2^ and 750 s/mm^2^. Only the 750 s/mm^2^ *b* value was able to accurately diagnosis liver fibrosis. Therefore, according to the formula above, the ADC value of the single *b* value was not sufficiently accurate to measure water molecules in liver tissue.

Yamada *et al.* [29] first reported IVIM-MRI technology in the liver. The IVIM technology was used with 4 *b* values (30, 300, 900, 1100 s/mm^2^); there was no significant difference between normal livers and those with cirrhosis. Luciani *et al.* [30] analyzed 37 cases (12 cases of patients with cirrhosis and 25 cases of healthy livers), and assessed the difference between livers with cirrhosis and normal livers by IVIM (*b* values: 0, 10, 20, 30, 50, 80, 100, 200, 400, 800 s/mm^2^). The ADC values of the liver cirrhosis group were significantly lower than those of the normal liver group (1.23 × 10^−3^ vs. 1.39 × 10^−3^ mm^2^/s).

Patel *et al.*[11] studied 16 cases without, and 14 cases with, liver cirrhosis, which adopted IVIM technology and chose 9 *b* values (0, 50, 100, 150, 200, 300, 500, 700, 1000 s/mm^2^). The ADC values and real values of water molecules were significantly lower in the liver cirrhosis group than those in the no cirrhosis group (1.41 × 10^−3^ vs. 1.73 × 10^−3^ mm^2^/s and 1.04 × 10^−3^ vs. 1.17 × 10^−3^ mm^2^/s). Although in recent years, IVIM-MRI technology was used in research of chronic liver disease with values both less and more than 200 s/mm^2^, in this study the values of ADC_total_ and ADC_0-400-600-800_ of the liver fibrosis group were significantly lower than those of the normal liver group, especially the ADC_0-400-600-800_ values. The reasons for the differences between the results of our study and those of Yamada *et al.* and Luciani *et al.* were the number of *b* values selected and the *b* value distribution, as well as our selected cases of hepatic fibrosis and its different stages.

With the increased progression of liver fibrosis, the accumulation of proteins in the extracellular matrix can also gradually increase. Our results verified that the mean values of ADC_total_ and ADC_0-400-600-800_ decreased as fibrosis scores increased. This data was consistent with the study by Taouli *et al.* [31] and Zeng Y [32], who confirmed a significant correlation between liver fibrosis staging and ADC values. For further comparison, we found significant differences between the subgroups F0-1 and F2-4, and between the subgroups F0-2 and F3-4, and between the subgroups F0-3 and F4 (*P* < 0.05) in liver fibrosis. In this study, we included 4 patients for both F1 and F4 groups because patients at F1 stage are hard to be detected due to absence of symptoms, while patients at F4 stage could be bleeding after liver biopsy and poor treatment by clinical medication. Clinically, high accuracy in the diagnosis of fibrosis with stage 2 or less could be acceptable for identifying candidates for treatment to prevent progression [1–3]. However, identifying fibrosis at stage 3 or greater is essential because patients with advanced fibrosis or cirrhosis should be screened for portal hypertension and hepatocellular carcinoma [33].

Our study had several limitations. First, the results were limited by the sample size, especially the small number of patients with fibrosis stage F1 and F4. A large number of patients in each fibrosis stage need to be compared in future study. Secondly, in this study, we analyzed various etiologies of liver fibrosis which could have a certain difference among diverse etiologies, thus we will study the etiology of liver fibrosis in our next step. Thirdly, we did not analyze the deposition of iron and fat in liver fibrosis and we do not know whether they would affect the values of ADC_total_ and ADC_0-400-600-800_. Fourthly, ADC values are composed of water diffusion and blood microcirculation perfusion, which are hard to be distinguished and could affect ADC values in liver fibrosis. Our future work will test IVIM parameters with biexponential model in liver fibrosis.

## MATERIALS AND METHODS

### Study design and patient population

From October 2012 to June 2013, thirty patients with chronic hepatitis were prospectively enrolled from outpatients and inpatients of the Department of Digestion in our hospital. All participants had an IVIM examination and conventional MRI. Among them, five patients were excluded from the study because of no liver biopsy, poor images, bad cooperation or other possible suspicious lesions found on conventional MRI findings. Finally, twenty-eight patients (23 males and 5 females, ages ranging from 25 to 73 years with a mean age of 43.8 ± 1.1 years) were enrolled into our study. Among the 28 patients, there were 18 cases of hepatitis B, one case of hepatitis C, one case of alcoholic hepatitis, and unknown causes in eight patients with elevated transaminase levels. All the patients had liver biopsies within one month of the MRI. Clinically, nine patients had slight abdominal distension and sixteen patients were asymptomatic. Twenty-five volunteers (15 males and 10 females, ages ranging from 25 to 57 years with a mean age of 38.9 ± 1.3 years who had no liver diseases or abnormal liver function) were used as the control group. The normality of volunteers was checked by biochemical testing of blood. All participants were given informed consent. All participants were given informed consent and the study was approved by the Ethics Committee of the People’s Hospital of Zhengzhou University.

### Examination and post processing

All participants were imaged with a 3.0T MR unit (GE Medical System, Discovery MR750, USA) with an 8-channel torso SD array body coil. Conventional MRI included axial T1-weighted spin echo (TR 180 ms, TE 2.1 ms), T2-weighted spin echo (TR 4286 ms, TE 88.1 ms), and coronal T2-weighted spin echo (TR 8571 ms, TE 88.8 ms). IVIM examination data were obtained adopting DW EPI with respiratory-triggered sequence (TR, 12 000 ms; TE, 62.1 ms; Scan matrix, 160 × 192; number of signals acquired, seven *b* values, 0, 50, 100, 200, 400, 600, 800 s/mm^2^; FOV, 360 × 324 mm; section thickness, 3 mm; and interstice gap, 1 mm). All regions of interest (ROIs) were manually positioned by two experienced radiologists who were in agreement with DW EPI diffusion images on the workstation for all *b* values. When we selected all the *b* values (0, 50, 100, 200, 400, 600, 800 s/mm^2^), we got the standard ADC value of the monoexponential model, namely ADC_total_. When we selected the four *b* values (0, 400, 600, 800 s/mm^2^), we got the standard ADC value of the monoexponential model, namely ADC_0-400-600-800_. Both values were calculated according to the formula [10, 11] of Sb/S0 = exp (bADC) respectively. ROIs (about 150 mm^2^) [12, 13] were positioned in the right posterior hepatic lobe, the right anterior hepatic lobe, the medial segment of the left lobe of each person, respectively. Care was taken to avoid large vessels, blurred regions, and any focal lesions. The values obtained from each person were calculated by averaging the three ROI measurements.

### Histopathology

Within 1 month of MRI, all patients with liver fibrosis were punctured. The stage of fibrosis was determined based on the histopathology of an 18-gauge ultrasound guided core biopsy liver sample [14–17], taken from the posterior lobe of the liver. Two experienced pathologists (with 15 years and 6 years of working experience in abdominal diagnosis) scored pathological specimens (Figure 1C and 1D) using the Knodell scoring system. The METAVIR semi-quantitative scoring system was used in the histopathologic determination of the stage of fibrosis. Fibrosis was staged on an F scale of 0–4, as follows: F0, indicating no fibrosis; F1, portal fibrosis without septa; F2, bridging fibrosis with few septa; F3, numerous septa without cirrhosis; and F4, early cirrhosis [18].

### Statistical analysis

The data were analyzed with the SPSS 16.0 software. Descriptive statistics were used for the study and the control groups. All data were presented as means ± SD. The LSD method of multiple comparison analysis of variance was used for calculating the mean ADC_total_ and ADC_0-400-600-800_ values obtained from the right posterior hepatic lobe, right anterior hepatic lobe and medial segment of the left lobe of the study group and the control group, respectively. ROC curves were used when calculating the AUC, sensitivity, specificity and 95% confidence intervals of the study and control groups. Positive predictive values (PPV) and negative predictive values (NPV) were calculated. To compare the mean values (ADC_total_ and ADC_0-400-600-800_) of the study and control groups, independent sample *t* tests and 95% confidence intervals were used. Spearman rank correlation analysis was conducted to examine the relationship among all the values and fibrosis stages. All values among the different fibrosis stages (subgroups F0-1 and F2-4, subgroups F0-2 and F3-4, subgroups F0-3 and F4) were compared in the study group by using independent sample *t* tests. A *P*-value < 0.05 was considered to indicate a significant difference.
